# The positive efficacy of dexmedetomidine on the clinical outcomes of patients undergoing renal transplantation: evidence from meta-analysis

**DOI:** 10.18632/aging.205296

**Published:** 2023-12-11

**Authors:** Shanshan Guo, Degong Jia, Xueqi Liu, Li Gao, Huaying Wang, Chaoyi Chen, Yonggui Wu

**Affiliations:** 1 Department of Nephropathy, The First Affiliated Hospital of Anhui Medical University, Hefei, Anhui 230022, PR China; 2 Department of Hepatobiliary Surgery, The Second Affiliated Hospital of Chongqing Medical University, Chongqing, PR China; 3 Center for Scientific Research of Anhui Medical University, Hefei, Anhui 230022, PR China

**Keywords:** renal transplantation, end-stage renal disease, dexmedetomidine, delayed graft function, meta-analysis

## Abstract

Introduction: Whether dexmedetomidine (DEX), an anesthetic adjuvant, can improve renal transplant outcomes is not clear.

Methods: We systematically identified clinical trials in which DEX was administered in renal transplantation (RT). On November 1, 2022, we searched The Cochrane Library, MEDLINE, EMBASE and https://www.clinicaltrials.gov/. The main outcomes were delayed graft function and acute rejection.

Results: A total of seven studies were included in the meta-analysis. The results showed that compared with the control, DEX significantly reduced the occurrence of delayed graft function (RR 0.76; 95% CI 0.60–0.98), short-term serum creatinine [postoperative day (POD) 2: (MD −22.82; 95% CI −42.01 – −3.64)] and blood urea nitrogen [POD 2: (MD −2.90; 95% CI −5.10 – −0.70); POD 3: (MD 2.07; 95% CI −4.12 – −0.02)] levels, postoperative morphine consumption (MD −4.27; 95% CI −5.92 – −2.61) and the length of hospital stay (MD −0.85; 95% CI−1.47 – −0.23). However, DEX did not reduce the risk of postoperative acute rejection (RR 0.75; 95% CI 0.45–1.23). The results of the subgroup analysis showed that country type, donor type, and average age had a certain impact on the role of DEX.

Conclusions: DEX may improve the short-term clinical outcome of RT and shorten the length of hospital stay of patients.

## INTRODUCTION

End-stage renal disease (ESRD) is a major public health problem with a high incidence and mortality rate [1]. Renal transplantation (RT) is recognized as the optimum treatment option for patients with ESRD, which has a higher survival rate and quality of life than dialysis and is generally more economical [2–4]. Delayed graft function (DGF) is an important factor affecting the efficacy of transplantation because it seriously deteriorates the likelihood of graft survival. Because of organ shortages, DGF is increasing due to the use of marginal kidneys. The incidence of DGF in living donors is approximately 4%–10%, and in deceased donors, it is approximately 20%–50% [5]. It is necessary to find appropriate drugs or technologies to promote the normal function of transplanted new organs.

Dexmedetomidine (DEX) is a highly effective and selective alpha-2 adrenergic receptor agonist, an effective auxiliary drug used to induce clinical anesthesia, with anti-anxiety, sedative and analgesic activities. Preclinical studies have shown that DEX can reduce sympathetic nerve tension and catecholamine levels, thereby reducing renal IRI [6–8]. Clinically, DEX has also shown a strong protective effect against a variety of acute kidney injuries (AKI) [9, 10]. Two meta-analyses further confirmed that perioperative infusion of DEX reduces the risk of AKI induced by cardiac surgery [11, 12]. Remarkably, our latest study identified the positive effects of DEX on liver transplantation [13]. Therefore, DEX is an attractive choice to improve the clinical prognosis of RT, but its application in renal transplant patients has yielded contradictory results [14–16].

To further study and clarify the effectiveness and safety of this convenient and economic intervention in RT, we conducted this meta-analysis. In addition, we aimed to assess which factors can affect the efficacy of DEX.

## RESULTS

### Summary of included studies

**Figure 1:**
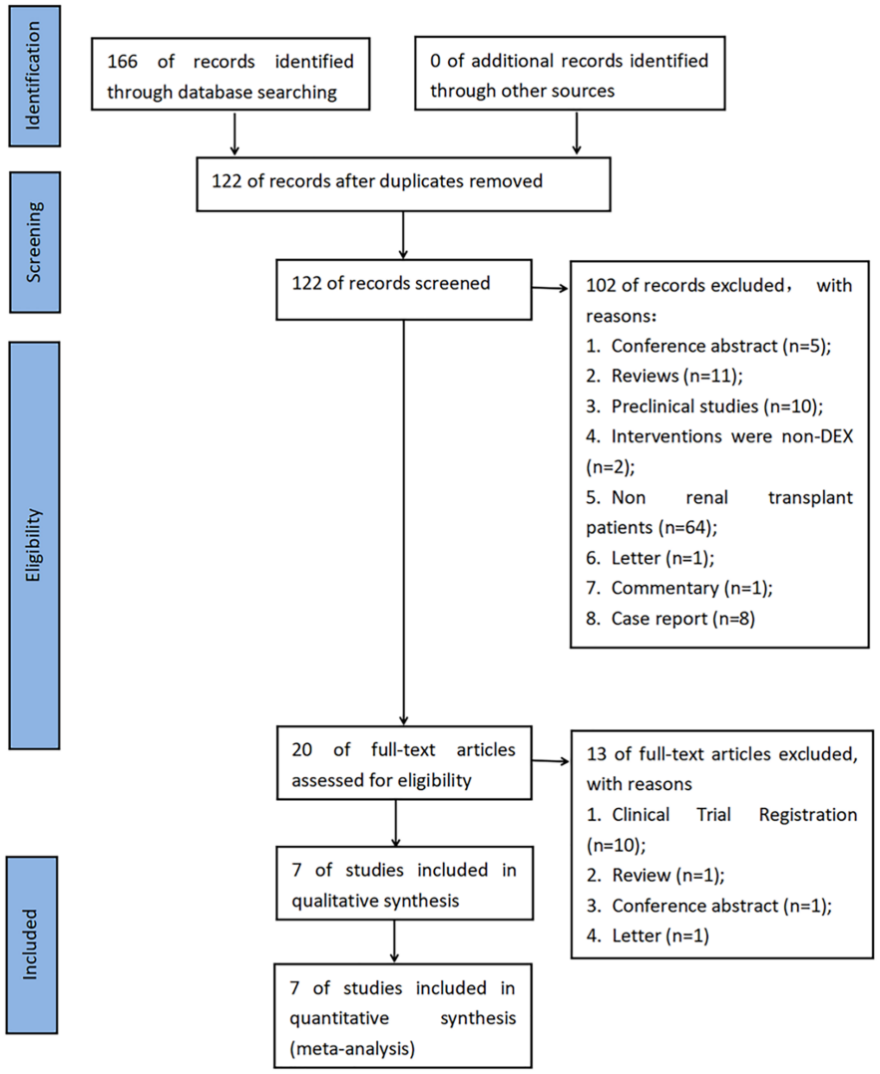
**PRISMA flow chart for the systematic review and the meta-analysis of literature retrieval and screening.** Abbreviation: DEX: dexmedetomidine.

A total of 166 articles were retrieved from the database. After removing the duplicate literature, 122 articles were reviewed, of which 115 articles were removed. After searching https://www.clinicaltrials.gov/, no new research was found. Finally, seven studies [14–20] were included in the meta-analysis, with a total of 1217 participants, 534 of whom received DEX. The combined search results are shown in Figure 1. Of the seven included studies, one was a retrospective cohort study [14], and the remaining were RCTs. Table 1 shows the basic characteristics of the included studies. Table 2 shows all the research results. Table 3 shows all the results of the subgroup analysis. Table 4 is the “Summary of findings” table.

**Table 1:** Characteristics of the included studies in this meta-analysis.

**Author, Year**	**No. of patients**		**Age**		**Female %**		**BMI**		**Intervention**	**Control**	**Follow up**	**Study type**
	DEX	Control	DEX	Control	DEX	Control	DEX	Control				
Jin Ha Park, 2021 [15]	51	52	49.94	48.35	54.90	55.77	N/S	N/S	dexmedetomidine 200μg was added to 0.9% saline in 50 mL and was administered at a rate of 0.4 μg/kg/hr, starting immediately after anesthesia induction and until the end of surgery.	0.9% saline in 50 ml was administered at a rate of 0.4 μg/kg/hr, starting immediately after anesthesia induction and until the end of surgery.	N/S	RCT
Jun Chen, 2020 [14]	315	465	51.90	52.50	38.41	31.61	27.60	27.40	intravenous infusion of dexmedetomidine (0.24 to 0.6 μg/kg/hr) initiated after induction of anesthesia and discontinued at the end of surgery.	nothing	N/S	retrospective cohort study
Peng Yang, 2020 [18]	19	19	38.50	41.60	36.84	31.58	23.10	23.80	an ultrasound-guided unilateral TAP block with 30 mL of 0.33% ropivacaine mixed with 1 μg/kg DEX, as well as morphine IV-PCA	an ultrasound-guided unilateral TAP block with 30 mL of 0.33% ropivacaine, as well as morphine IV-PCA	N/S	RCT
Sunder Negi, 2014 [17]	30	30	34.33	35.80	16.67	26.67	N/S	N/S	0.5 μg/kg dexmedetomidine infusion diluted to 20 ml intravenous solution over 10 minutes before induction of anesthesia, followed by 0.5 μg/kg dexmedetomidine in combination with 5 ml of 0.25% ropivacaine by epidural route (total volume 8 ml).	1 μg/kg fentanyl infusion diluted to 20 ml intravenous fluid over 10 minutes before induction of anesthesia and 1 μg/kg fentanyl in combination with 5 ml of 0.25% ropivacaine (total volume 8 ml) via epidural route after insertion of epidural catheter	N/S	RCT
Xi-sheng Shan, 2022 [16]	56	55	43.50	43.30	35.71	49.09	21.80	21.10	24-hour perioperative dexmedetomidine intravenous infusion (0.4 μg/kg/h intraoperatively and 0.1 μg/kg/h postoperatively)	24-hour perioperative saline intravenous infusion (0.4 μg/kg/h intraoperatively and 0.1 μg/kg/h postoperatively)	1y	RCT
Yin-Chin Wang, 2022 [20]	30	30	43.36	43.36	40.00	33.33	N/S	N/S	0.1–0.7 mg/kg/h dexmedetomidine infusion until 2 h after surgery	nothing	N/S	RCT
Zhenzhen Liu, 2022 [19]	33	32	40.76	42.59	30.30	18.75	24.15	23.20	an initial loading dose of 0.6 μg/kg Dex intravenously for 15 min before anaesthesia induction, followed by a rate of 0.4 μg/kg/h until 30 min after kidney reperfusion	saline	3m	RCT

Abbreviations: DEX: dexmedetomidine; N/S: not stated.

**Table 2:** Summary of the results of the meta-analysis of the efficacy of DEX in RT.

**Outcomes**	**No. of included studies**	**Total number of DEX and control**	**Heterogeneity**	**Effect estimation**	***P*-value**
Delayed graft function	4	455 of DEX and 604 of control	I^2^ 0%	RR 0.76; 95% CI: 0.60–0.98	0.03
Acute rejection	3	422 of DEX and 572 of control	I^2^ 0%	RR 0.75; 95% CI: 0.45–1.23	0.25
Creatinine μmol/L					
POD 1	6	212 of DEX and 215 of control	I^2^ 72%	MD −43.65; 95% CI: −102.88–15.58	0.15
POD 2	4	163 of DEX and 166 of control	I^2^ 0%	MD −22.82; 95% CI −42.01 – −3.64	0.02
POD 3	4	163 of DEX and 166 of control	I^2^ 0%	MD −14.21; 95% CI: −30.50–2.07	0.09
POD 7	4	451 of DEX and 601 of control	I^2^ 0%	MD −9.79; 95% CI: −23.93–4.34	0.17
1m	3	399 of DEX and 546 of control	I^2^ 0%	MD −6.43; 95% CI: −22.32–9.46	0.43
3m	3	393 of DEX and 537 of control	I^2^ 5%	MD −4.87; 95% CI: −14.26–4.53	0.31
BUN mmol/L					
POD 1	4	105 of DEX and 108 of control	I^2^ 46%	MD −1.20; 95% CI: −2.92–0.52	0.17
POD 2	2	56 of DEX and 59 of control	I^2^ 16%	MD −2.90; 95% CI: −5.10 – −0.70	0.01
POD 3	2	56 of DEX and 59 of control	I^2^ 0%	MD −2.07; 95% CI: −4.12 – −0.02	0.05
Duration of surgery	5	168 of DEX and 166 of control	I^2^ 45%	MD −3.93; 95% CI: −8.26–0.40	0.08
Postoperative morphine consumption	2	49 of DEX and 49 of control	I^2^ 0%	MD −4.27; 95% CI: −5.92 – −2.61	<0.00001
Length of hospital stay	4	455 of DEX and 604 of control	I^2^ 10%	MD −0.85; 95% CI: −1.47 – −0.23	0.007

Abbreviations: BUN: blood urea nitrogen; DEX: dexmedetomidine; POD: postoperative day.

**Table 3:** Summary of the results of the subgroup analysis of DEX in RT.

**Outcome**	**Subgroup**	**No. of studies**	**Population size**	**Effect estimation (95% CI)**	**I^2^ statistic (%)**
Delayed graft function	Retrospective	1	780	0.34–1.01	0
	Prospective	3	279	0.62–1.08	–
	Developed	2	883	0.62–1.08	0
	Non-developed	2	176	0.32–0.99	0
	Deceased donor	2	176	0.32–0.99	0
	Living donor	1	103	0.07–15.87	–
	>44 years old	2	883	0.62–1.08	0
	<44 years old	2	176	0.32–0.99	0
Acute rejection	Retrospective	1	780	0.16–1.51	–
	Prospective	2	214	0.49–1.51	0
	Developed	2	883	0.43–1.34	2
	Non-developed	1	111	0.24–2.08	–
	Deceased donor	1	111	0.24–2.08	–
	Living donor	1	103	0.49–1.81	–
	>44 years old	2	883	0.43–1.34	2
	<44 years old	1	111	0.24–2.08	–
Duration of surgery (min)	Prospective	5	334	−8.26–0.40	45
	Non-developed	5	334	−8.26–0.40	45
	Deceased donor	2	176	−11.69–11.12	69
	Living donor	1	60	−9.59–1.59	–
	<44 years old	5	334	−8.26–0.40	45
Creatinine (μmol/L) POD1	Prospective	6	427	−102.88–15.88	72
	Developed	1	103	−115.52 – −10.01	–
	Non-developed	5	324	−114.59–36.32	72
	Deceased donor	2	166	− 91.98–106.82	0
	Living donor	2	163	−117.79 – −28.15	0
	>44 years old	1	103	−115.52 – −10.01	–
	<44 years old	5	324	−114.59–36.32	72
Creatinine (μmol/L) POD2	Prospective	4	329	−42.01 – −3.64	0
	Developed	1	103	−36.57–6.51	–
	Non-developed	3	226	−94.84 – −10.53	0
	Deceased donor	2	166	−149.67–84.76	0
	Living donor	1	103	−36.57–6.51	–
	>44 years old	1	103	−36.57–6.51	–
	<44 years old	3	226	−94.84 – −10.53	0
Creatinine (μmol/L) POD3	Prospective	4	329	−30.50–2.07	0
	Developed	1	103	−23.95–16.88	–
	Non-developed	3	226	−59.92 – −5.89	0
	Deceased donor	2	166	−115.45–82.64	0
	Living donor	1	103	−23.95–16.88	–
	>44 years old	1	103	−23.95–16.88	–
	<44 years old	3	226	−59.92 – −5.89	0
Creatinine (μmol/L) POD7	Retrospective	1	778	−61.77–26.41	–
	Prospective	3	274	−23.81–6.03	0
	Developed	2	881	−25.05–21.55	0
	Non-developed	2	171	−32.25–3.30	0
	Deceased donor	1	111	−56.83–33.97	–
	Living donor	1	103	−23.02–31.86	–
	>44 years old	2	881	−25.05–21.55	0
	<44 years old	2	171	−32.55–3.30	0
Creatinine (μmol/L) 1 m	Retrospective	1	773	−27.70–10.02	–
	Prospective	2	172	−30.02–28.98	0
	Developed	1	773	−27.70–10.02	–
	Non-developed	2	172	−30.02–28.98	0
	Deceased donor	2	172	−30.02–28.98	0
	>44 years old	1	773	−27.70–10.02	–
	<44 years old	2	172	−30.02–28.98	0
Creatinine (μmol/L) 3 m	Retrospective	1	766	−13.88–13.88	–
	Prospective	2	164	−21.74–3.78	19
	Developed	2	868	−15.14–3.90	16
	Non-developed	1	61	−34.98–81.00	–
	Deceased donor	1	61	−34.98–81.00	–
	Living donor	1	103	−23.69–2.47	–
	>44 years old	2	868	−15.14–3.90	16
	<44 years old	1	61	−34.98–81.00	–
BUN (mmol/L) POD1	Prospective	4	213	−2.92–0.52	46
	Non-developed	4	213	−2.92–0.52	46
	Deceased donor	1	55	−2.03–4.58	–
	Living donor	1	60	−9.53–0.35	–
	<44 years old	4	213	−2.92–0.52	46
BUN (mmol/L) POD2	Prospective	2	115	−5.10 – −0.07	16
	Non-developed	2	115	−5.10 – −0.07	16
	Deceased donor	1	55	−5.21–377	–
	<44 years old	2	115	−5.10 – −0.07	16
BUN (mmol/L) POD3	Prospective	2	115	−4.12 – −0.02	0
	Non-developed	2	115	−4.12 – −0.02	0
	Deceased donor	1	55	−5.35–6.27	–
	<44 years old	2	115	−4.12 – −0.02	0
Length of hospital stay (days)	Retrospective	1	780	−1.51 – −0.09	–
	Prospective	3	279	−2.27–0.24	38
	Developed	2	883	−1.43 – −0.06	0
	Non-developed	2	176	−2.73–0.12	60
	Deceased donor	2	176	−2.73–0.12	60
	Living donor	1	103	−2.65–2.65	–
	>44 years old	2	883	−1.43 – −0.06	0
	<44 years old	2	176	−2.73–0.12	60
Morphine consumption (mg)	Prospective	2	98	−5.92 – −2.61	0
	Non-developed	2	98	−5.92 – −2.61	0
	Living donor	1	38	−5.94 – −2.08	–
	<44 years old	2	98	−5.92 – −2.61	0

Abbreviations: BUN: blood urea nitrogen; POD: postoperative day.

**Table 4:** Summary of findings table.

**Outcomes**	Illustrative comparative risks* (95% CI)		**Relative effect (95% CI)**	**No of Participants (studies)**	**Quality of the evidence (GRADE)**	**Comments**
	**Assumed risk control**	**Corresponding risk dexmedetomidine**				
**Creatinine-POD1**	The mean creatinine-pod1 ranged across control groups from **266.084 to 752.35 μmol/L**	The mean creatinine-pod1 in the intervention groups was **43.65 lower** (102.88 lower to 15.58 higher)		427 (6 studies)	⊕⊕⊕⊝ **moderate**^1^	
**Creatinine-POD2**	The mean creatinine-pod2 ranged across control groups from **126.412 to 571.914 μmol/L**	The mean creatinine-pod2 in the intervention groups was **22.82 lower** (42.01 to 3.64 lower)		329 (4 studies)	⊕⊕⊕⊕ **high**	
**Creatinine-POD3**	The mean creatinine-pod3 ranged across control groups from **108.732 to 476.707 μmol/L**	The mean creatinine-pod3 in the intervention groups was **14.21 lower** (30.5 lower to 2.07 higher)		329 (4 studies)	⊕⊕⊕⊕ **high**	
**Creatinine-POD7**	The mean creatinine-pod7 ranged across control groups from **93.704 to 164.77 μmol/L**	The mean creatinine-pod7 in the intervention groups was **8.89 lower** (23.81 lower to 6.03 higher)		274 (3 studies)	⊕⊕⊕⊕ **high**	
**Creatinine-POD7-1**	The mean creatinine-pod7-1 ranged across control groups from **380.12 to 380.12 μmol/L**	The mean creatinine-pod7-1 in the intervention groups was **17.68 lower** (61.77 lower to 26.41 higher)		778 (1 study)	⊕⊕⊝⊝ **low**	
**DGF**	**194 per 1000**	**113 per 1000** (66 to 196)	**RR 0.58** (0.34 to 1.01)	279 (3 studies)	⊕⊕⊕⊕ **high**	
**DGF-1**	**237 per 1000**	**194 per 1000** (147 to 255)	**RR 0.82** (0.62 to 1.08)	780 (1 study)	⊕⊕⊝⊝ **low**	
**BUN-POD1**	The mean bun-pod1 ranged across control groups from **16.9 to 26.04 mmol/L**	The mean bun-pod1 in the intervention groups was **1.2 lower** (2.92 lower to 0.52 higher)		213 (4 studies)	⊕⊕⊕⊝ **moderate**^1^	
**BUN-POD2**	The mean bun-pod2 ranged across control groups from **12.0 to 22.64 mmol/L**	The mean bun-pod2 in the intervention groups was **2.9 lower** (5.1 to 0.7 lower)		115 (2 studies)	⊕⊕⊕⊕ **high**	
**BUN-POD3**	The mean bun-pod3 ranged across control groups from **11.02 to 24.41 mmol/L**	The mean bun-pod3 in the intervention groups was **2.07 lower** (4.12 to 0.02 lower)		115 (2 studies)	⊕⊕⊕⊕ **high**	
**Acute rejection**	**196 per 1000**	**169 per 1000** (96 to 296)	**RR 0.86** (0.49 to 1.51)	214 (2 studies)	⊕⊕⊕⊕ **high**	
**Acute rejection-1**	**26 per 1000**	**13 per 1000** (4 to 39)	**RR 0.49** (0.16 to 1.51)	780 (1 study)	⊕⊕⊝⊝ **low**	

**Patient or population:** patients undergoing renal transplantation

**Intervention:** dexmedetomidine

**Comparison:** placebo or non-dexmedetomidine

^*^The basis for the **assumed risk** (e.g., the median control group risk across studies) is provided in footnotes. The **corresponding risk** (and its 95% confidence interval) is based on the assumed risk in the comparison group and the **relative effect** of the intervention (and its 95% CI).

**Abbreviations: BUN:** blood urea nitrogen; **DGF:** delayed graft function; **CI:** Confidence interval; **RR:** Risk ratio.

GRADE Working Group grades of evidence

**High quality:** Further research is very unlikely to change our confidence in the estimate of effect.

**Moderate quality:** Further research is likely to have an important impact on our confidence in the estimate of effect and may change the estimate.

**Low quality:** Further research is very likely to have an important impact on our confidence in the estimate of effect and is likely to change the estimate.

**Very low quality:** We are very uncertain about the estimate.

^1^Downgraded one level: heterogeneity exists between studies.

The overall risk of bias of the included studies was low. Supplementary Figure 1 shows the quality evaluation results of each included study. The quality risk assessment of these studies concluded that all the studies were of good quality.

### Primary outcome

#### Delayed graft function

**Figure 2:**
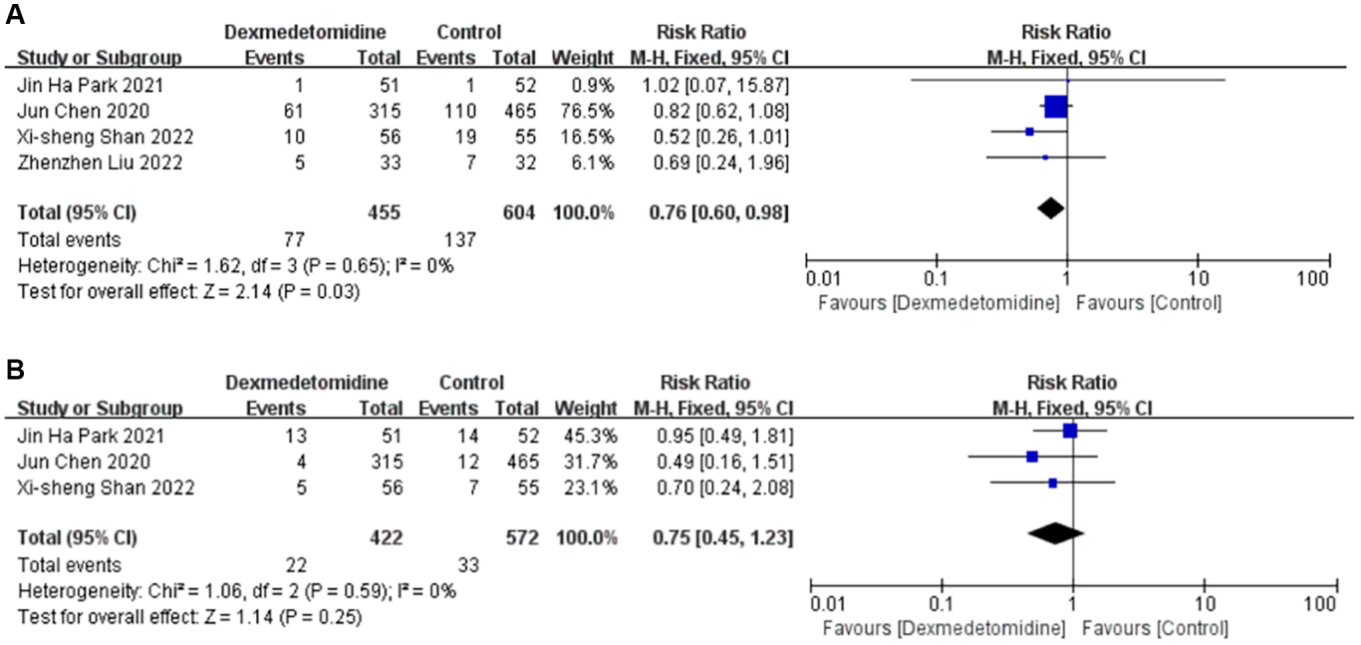
Forest plots of the effects of DEX on DGF (**A**) and acute rejection (**B**). Abbreviations: DEX: dexmedetomidine; DGF: delayed graft function.

Four studies [14–16, 19] described the incidence of DGF in 1059 patients, 455 of whom received DEX. Meta-analysis showed that the perioperative use of DEX could significantly decrease the occurrence of DGF (RR 0.76; 95% CI 0.60–0.98; *P* = 0.03; I2 = 0%; low certainty evidence) (Figure 2A). The results are also confirmed in subgroups such as non-developed countries and deceased donors (Table 3).

#### Acute rejection

Three studies [14–16] described the incidence of acute rejection in 994 patients, 422 of whom were treated with DEX. Meta-analysis showed that DEX did not significantly lower the occurrence of acute rejection (RR 0.75; 95% CI 0.45–1.23; *P* = 0.25; I2 = 0%; low certainty evidence) (Figure 2B).

### Secondary outcome

#### Postoperative renal function

**Figure 3:**
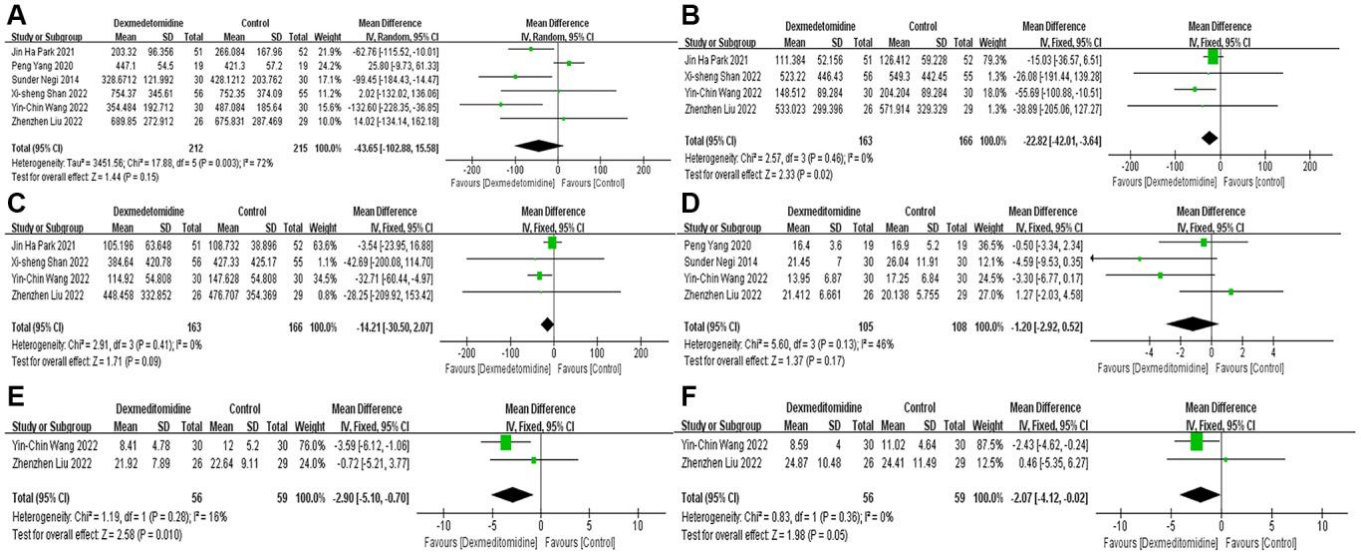
Forest plots of the effects of DEX on creatinine (POD 1-3) (**A**–**C**) and BUN (POD 1-3) (**D**–**F**). Abbreviations: BUN: blood urea nitrogen; DEX: dexmedetomidine; POD: postoperative day.

We assessed serum creatinine (μmol/L), blood urea nitrogen (BUN) (mmol/L) and estimated glomerular filtration rate (eGFR) (ml/min/1.73m^2^) at different times after surgery to reflect postoperative renal function. Six studies [15–20] recorded the influence of DEX on the postoperative day (POD) 1 creatinine level and found no significant difference between the two groups (MD −43.65; 95% CI −102.88–15.58; *P* = 0.15; I2 = 72%; moderate certainty evidence) (Figure 3A). Four studies [15, 16, 19, 20] described the creatinine level on POD 2 and showed that DEX significantly reduced the serum creatinine level on POD 2 (MD −22.82; 95% CI −42.01 – −3.64; *P* = 0.02; I2 = 0%; high certainty evidence) (Figure 3B). Four studies [15, 16, 19, 20] recorded the creatinine level on POD 3, and DEX had no significant impact on the level of creatinine on POD 3 (MD −14.21; 95% CI −30.50–2.07; P = 0.09; I2 = 0%; high certainty evidence) (Figure 3C). In addition, we did not find that DEX had a significant impact on the level of creatinine on the seventh day (MD −9.79; 95% CI −23.93–4.34; *P* = 0.17; I2 = 0%; low certainty evidence), one month (MD −6.43; 95% CI −22.32–9.46; *P* = 0.43; I2 = 0%) or three months (MD −4.87; 95% CI −14.26–4.53; *P* = 0.31; I2 = 5%) after the operation (Supplementary Figure 2).

The change in BUN level was consistent with that of creatinine. Compared with the control, although DEX had no significant effect on the BUN level on POD 1 (MD −1.20; 95% CI −2.92–0.52; *P* = 0.17; I2 = 46%; moderate certainty evidence) (Figure 3D), it significantly reduced the BUN level on POD 2 (MD −2.90; 95% CI −5.10 – −0.70; *P* = 0.01 I2 = 16%; high certainty evidence) (Figure 3E) and POD 3 (MD −2.07; 95% CI −4.12 – −0.02; *P* = 0.05; I2 = 0%; high certainty evidence) (Figure 3F).

Postoperative eGFR was also examined. Due to the lack of early postoperative eGFR data, we only analyzed the postoperative eGFR levels at one month and three months. The results showed that compared with the control group, DEX had no significant effect on one-month (MD 0.66; 95% CI −2.46–3.77; *P* = 0.68 I2 = 0%) or three-month (MD −0.39; 95% CI −3.28–2.50; *P* = 0.79 I2 = 0%) eGFR after surgery (Supplementary Figure 3).

Therefore, we concluded that DEX might improve early postoperative renal function, and this effect may be related to the country type and the average age of people (Table 3).

#### Duration of surgery

**Figure 4:**
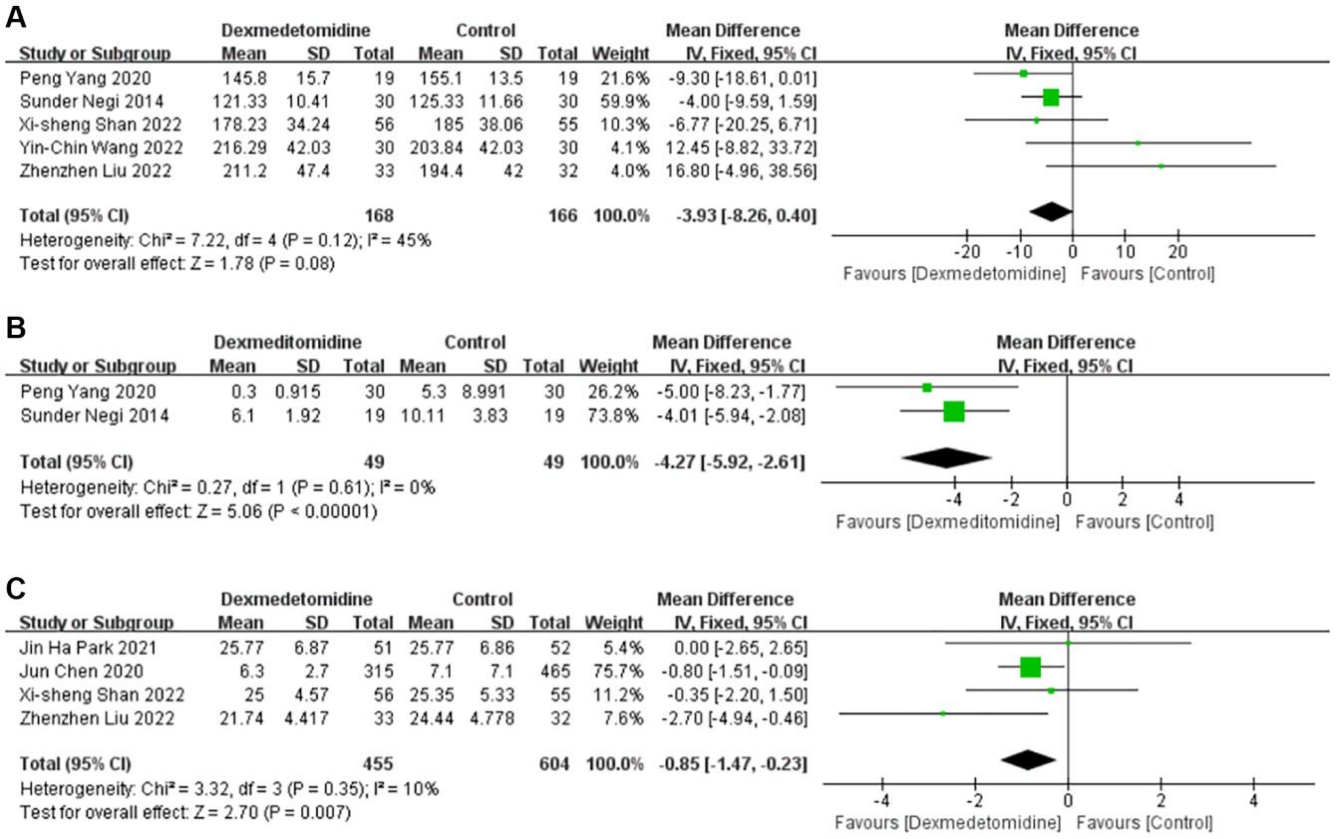
Forest plots of the effects of DEX on the duration of surgery (**A**), postoperative morphine consumption (**B**) and LOS (**C**). Abbreviations: DEX: dexmedetomidine; LOS: length of hospital stay.

Five studies [16–20] recorded the duration of surgery (min) in 334 patients. Compared with the control group, DEX had no significant impact on the duration of surgery (MD −3.93; 95% CI −8.26–0.40; *P* = 0.08; I2 = 45%) (Figure 4A).

#### Postoperative morphine consumption

In three studies [17, 18, 20], morphine was used after surgery, but only two studies [17, 18] recorded specific consumption. The results showed that DEX significantly reduced postoperative morphine consumption (MD −4.27; 95% CI −5.92 – −2.61; *P* < 0.00001; I2 = 0%) (Figure 4B).

#### Length of hospital stay

Four studies [14–16, 19] described the Length of hospital stay [LOS (d)] after surgery. Meta-analysis showed that DEX significantly reduced the LOS (MD −0.85; 95% CI −1.47 – −0.23; *P* = 0.007; I2 = 10%) (Figure 4C). Subgroup analysis showed that study type, country type and average age of the population could have affected this effect (Table 3).

## DISCUSSION

Our meta-analysis indicated that DEX use reduced the occurrence of DGF and both the creatinine and BUN levels in the early postoperative period. In addition, the participants in the DEX group had less postoperative morphine consumption and a shorter LOS. However, we did not find that DEX significantly affected the duration of surgery or the risk of postoperative acute rejection.

Our study found that DEX can reduce the incidence of DGF, which is of great significance. DGF is one of the main early complications after RT and is a unique form of AKI during RT [21]. DGF is independently associated with poor graft function, high rejection risk, and poor graft and patient survival, and a >50% DGF risk was even associated with a 2-fold increased risk of graft failure [22, 23]. In addition, this finding is consistent with the fact that DEX reduces the creatinine and BUN levels in the early postoperative period. It has been reported that an increase of 0.3 mg/dL in serum creatinine level is associated with a two-fold increase in the risk of long-term graft loss [24]. Slight improvement in early renal allograft function has great advantages for future prognosis. It is easy to understand the positive effect of DEX on early renal function after RT. Alpha-2 adrenergic receptors are widely distributed in proximal and distal tubules and the vascular system around renal tubules. Its activation can induce vasodilation by regulating endothelial nitric oxide synthase, thereby increasing the urine volume and improving renal allograft function [25]. Overall, the positive effect of DEX on early postoperative graft function may be of great significance for the long-term prognosis of patients. Regrettably, the effects of DEX on the duration of DGF have not been further reported in relevant studies. Not only does DGF significantly affect long-term prognosis, but the duration of DGF has also been proven to be an independent predictor of long-term graft function and survival [26, 27]. Future studies should disclose the effect of DEX on DGF duration.

DEX has immunomodulatory and anti-inflammatory properties, which play an important role in the occurrence and development of acute rejection [28]. At the same time, the reduction in DGF can also reduce the incidence of acute rejection [29]. However, our study did not observe a significant difference in the effect of DEX on acute rejection, which may be because the three studies that had relatively limited sample sizes been unable to detect this difference. In the future, more and larger clinical trials are needed to determine whether DEX affects the occurrence of postoperative acute rejection.

Patients receiving RT often suffer from moderate to severe pain after surgery. Effective postoperative analgesia promotes the early rehabilitation of patients. At present, opioids are the main drugs used for postoperative analgesia, but they can easily lead to gastrointestinal function inhibition, nausea, vomiting, respiratory inhibition and infection [30, 31]. In addition, the postoperative opioid dosage in patients was also found to be correlated with mortality and transplant failure within one year after surgery [32]. Our study found that DEX can reduce postoperative morphine consumption. This indicates that DEX has the potential to reduce the postoperative opioid dosage and the occurrence of related complications.

The results showed that DEX significantly reduced the LOS. However, the overall reduction time is less than 1 day. The results may or may not be clinically significant. Thus, we need more clinical data to determine this role of DEX.

At the same time, we are committed to finding the factors influencing the effect of DEX on RT. Considering factors such as country type, research type, donor type and average age, we found that in deceased donors, non-developed countries and people younger than 44 years old, DEX has a more stable effect on reducing the incidence of DGF and improving early postoperative renal function. In addition, we have not found that different research types have a significant impact on the efficacy of DEX. However, the number of studies for each result is limited, and more data are needed to further determine the impact of these factors on the efficacy of DEX. Moreover, all patients included in this meta-analysis received a relatively low dose of DEX infusion (0.1–0.8 mg/kg/h). Although DEX will not cause bradycardia and hypotension at this rate, the protective effect of DEX is dose dependent [33]. It is uncertain whether a higher infusion rate has better clinical effects during RT. Thus, it is necessary to draw a dose-response curve, measure plasma DEX concentration and compare the indexes at various dosages of DEX. Moreover, some studies [15] did not use the initial loading dose, and the plasma concentration of DEX may not have been high enough, so the therapeutic efficacy was not good. In addition, DEX is more effective when administered before renal IRI than after it [6, 34]. The current research emphasizes only the importance of intervention in the recipient, but the donor kidney has already suffered an ischemic injury before transplantation. Whether DEX in donors, especially living donors or organ preservation, has better efficacy needs further study.

### Strengths and limitations

To the best of our knowledge, this is the first meta-analysis summarizing the efficacy of DEX in RT. Compared with any single study, this study provides more accurate measurement results through strict meta-analysis, which provides a basis for the treatment of RT with DEX. Of course, this article also has some limitations. First, the meta-analysis is a descriptive secondary analysis, and its results are prone to bias due to the shortcomings of the methodology and the heterogeneity of the included studies. Considering the existence of heterogeneity, we performed subgroup analysis for factors including country type, donor type, and average age. However, due to the limited number of studies, there are some other factors that may affect the outcome, such as variation in the doses or practice of how DEX was used, which cannot be further explored. More research and more attempts to use DEX in RT will help to solve this problem. Next, of the included studies, the study [14] with the largest sample size was a single-center, retrospective study. The chief anesthesiologist is responsible for deciding to use DEX in anesthesia according to his or her judgment, which inevitably leads to heterogeneity, thus increasing the risk of making Type I and Type II mistakes. Third, only three studies described graft and patient survival, and the follow-up time ranged from 1 month to 1 year. We could not summarize them to judge the impact of DEX on survival or mortality. Fourth, this study lacked long-term follow-up data of DEX on RT. Since most of the included studies were published after 2020, only one study [16] had a follow-up period of up to one year. We lack sufficient data to judge the impact of DEX on the long-term clinical outcome of RT, so there are still uncertainties regarding the long-term efficacy and safety of DEX on RT. Here, we call on these teams to conduct more continuous disease surveillance follow-up and further reporting for the time after RT. Finally, the characteristics of the patients and the transplant center will also have an impact on the results, such as the basic disease of the patient, the volume of the transplant center, and the surgical and postoperative nursing skills. We acknowledge the existence of this unavoidable heterogeneity.

## CONCLUSION

This study indicated that the perioperative infusion of DEX improved early postoperative graft function and reduced the LOS of renal transplant patients. DEX, as a low-cost and low-side-effect intervention, may be a promising renal allograft protection intervention. However, some of the included studies have limited sample sizes and significant heterogeneity. Thus, the quality, consistency and design of future randomized controlled trials of DEX in RT need to be improved, especially with patient-centered outcomes, to draw reliable conclusions.

## MATERIALS AND METHODS

This study is a post hoc analysis of previous studies of DEX in RT. This systematic review and meta-analysis were conducted according to PRISMA guidelines [35] and registered in PROSPERO (CRD42022372149).

### Search strategy

On November 1, 2022, we searched the Cochrane Library, MEDLINE, EMBASE and the clinical trial registration platform https://www.clinicaltrials.gov/. See Supplementary Table 1 for the complete search strategy. The search was not restricted by any characteristics. The results were imported into Endnote X9 for further filtering.

### Selection criteria

We used Endnote X9 to remove duplicate articles, and two independent reviewers (Shanshan Guo, Degong Jia) used a two-stage screening method to screen the remaining articles. We recorded the number of unqualified articles and the reasons for the title/abstract and full-text screening stage and manually identified the references finally included in the research and related reviews to ensure a more comprehensive search. Disagreements between the two reviewers were resolved through discussion or by the participation of a third reviewer (Yonggui Wu).

We formulated inclusion/exclusion criteria before the literature search. We included the original article related to kidney transplantation and kidney-pancreas transplantation. The study must have included DEX as the intervention measure and placebo or non-DEX as the control to meet the inclusion criteria. We excluded relevant studies that did not include the results required for this study.

### Research outcomes

We predefined the research results before the meta-analysis. The primary outcomes were DGF and acute rejection. DGF was defined as the need for dialysis during the first week after RT [36]. Secondary outcomes included postoperative renal function, duration of surgery, LOS, and postoperative morphine consumption (mg).

### Data extraction and quality assessment

One reviewer used standardized data extraction forms to extract the data included in the study, and the other reviewer checked the extracted data. The data are summarized as the mean ± standard deviation of continuous variables and the positive and total events of dichotomous variables. The preexisting formula is used for the conversion of values, in which the mean and standard deviation are estimated according to the median and range using the formula proposed by Wan et al. [37]. We tried to contact the original author to obtain missing data.

The quality of randomized controlled trials was evaluated using the Cochrane risk assessment tool [38]. Retrospective cohort studies were evaluated by the Newcastle Ottawa Quality Assessment scale. Research quality was not considered as the inclusion/exclusion criterion.

### Data synthesis and analysis

We used Review Manager version 5.3 for data synthesis and analysis. For dichotomous variables, the results are expressed by the 95% CI and RR value. For continuous variables, the MD and 95% CI were used for expression, and forest plots were used to visualize the results.

To avoid the interference of multiple comparisons on the efficacy judgment of DEX, we changed the traditional *P* value. We used 0.05 divided by the mean of 1 (no adjustment) and the number of main results (Bonferroni adjustment) to obtain multiple adjusted *P* values. That is, when there was a main result, *P* = 0.05; for the two main results, *P* = 0.033. Therefore, when analyzing the primary outcome, we considered a *P* value of 0.033 (calculated by dividing 0.05 by ((2 + 1)/2)) or less to be statistically significant. For the secondary outcome, we considered a *P* value of 0.02 (calculated by dividing 0.05 by ((4 + 1)/2)) or less to be statistically significant [39]. In the above equation, 2 represents two primary outcomes (DGF and acute rejection), and 4 represents four secondary outcomes (duration of surgery, postoperative renal function, LOS, and postoperative morphine consumption).

The chi^2^ and I^2^ tests were used to evaluate the statistical heterogeneity among the studies. When PHeterogeneity <0.1 or I2 > 50%, the heterogeneity between the studies was considered. Data were summarized by the random effect model when there was heterogeneity among the studies; otherwise, the fixed effect model was used [40]. Subgroup analysis of the primary and secondary outcomes of studies was conducted with reference to country type (developed or non-developed), research type (retrospective or prospective), donor type (deceased donor or living donor), and average age (>44 years old or <44 years old).

### “Summary of findings” table

We summarized the main results in the “Summary of findings” table. This table contains information on the quality of the evidence and the impact of the intervention. The GRADE approach was used to overall grade the evidence associated with each main outcome [41]. The five considerations (study limitations, consistency of effect, imprecision, indirectness and publication bias) were taken into account to assess the quality of relevant evidence.

### Data availability statement

All data generated or analyzed during this study are included in this Article and its Supplementary Material Files. Further enquiries can be directed to the corresponding author.

## Supplementary Materials

Supplementary Figures

Supplementary Table 1
