# Supplementary material for: The positive efficacy of dexmedetomidine on the clinical outcomes of patients undergoing renal transplantation: evidence from meta-analysis
Source: Aging (Albany NY). 2023 Dec 11;15(23):14192–209. doi: 10.18632/aging.205296 (PMC10756127; doi:10.18632/aging.205296)
Supplement: Supplementary Figures [file aging-15-205296-s001.pdf]

SUPPLEMENTARY FIGURES

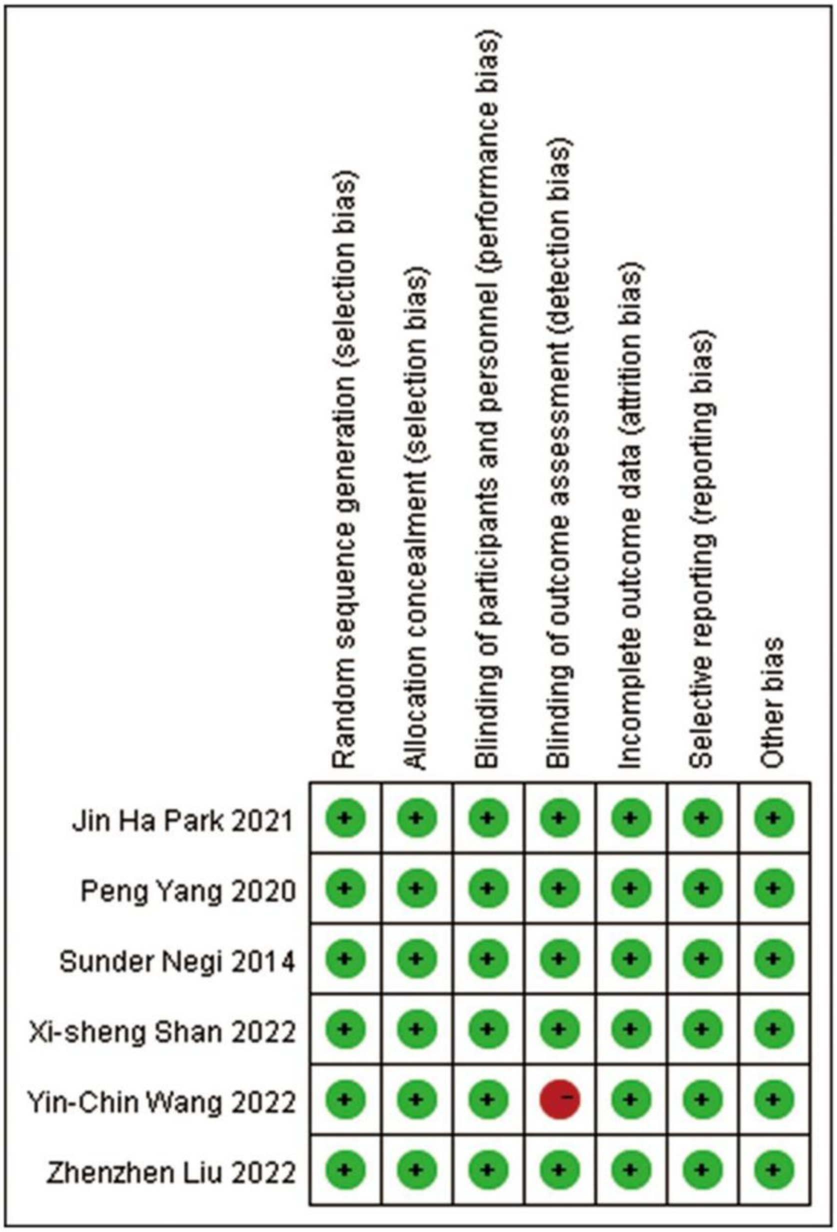

Cohort studies	Selection				Comparability	Outcome		
	Representativeness of the exposed cohort	Selection of the non exposed cohort	Ascertainment of exposure	Demonstration that outcome of interest was not present at start of study	Comparability of cohorts on the basis of the design or analysis	Assessment of the outcome	Was follow-up long enough for outcomes to occur	Adequacy of follow up of cohorts
Jun Chen ,2020	*	*	*	*	*	*		*

Supplementary Figure 1. Quality evaluation results of the included studies.

A

Study or Subgroup	Dexmedetomidine Mean	SD	Total	Control Mean	SD	Total	Weight	Mean Difference IV, Fixed, 95% CI
Jin Ha Park 2021	98.124	76.908	51	93.704	64.532	52	26.5%	4.42 [-23.02, 31.86]
Jun Chen 2020	362.44	300.56	314	380.12	318.24	464	10.3%	-17.68 [-61.77, 26.41]
Xi-sheng Shan 2022	153.34	108.66	56	164.77	133.83	55	9.7%	-11.43 [-56.83, 33.97]
Yin-Chin Wang 2022	103.428	41.548	30	118.456	34.476	30	53.5%	-15.03 [-34.35, 4.29]
**Total (95% CI)**			**451**			**601**	**100.0%**	**-9.79 [-23.93, 4.34]**

Heterogeneity: Chi² = 1.44, df = 3 (P = 0.70); I² = 0%
Test for overall effect: Z = 1.36 (P = 0.17)

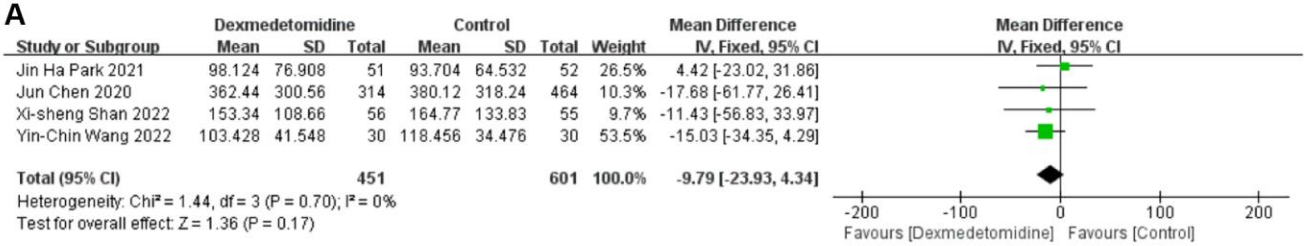

B

Study or Subgroup	Dexmedetomidine Mean	SD	Total	Control Mean	SD	Total	Weight	Mean Difference IV, Fixed, 95% CI
Jun Chen 2020	194.48	123.76	311	203.32	141.44	462	71.0%	-8.84 [-27.70, 10.02]
Xi-sheng Shan 2022	125.528	81.328	56	138.788	122.876	55	16.7%	-13.26 [-52.10, 25.58]
Zhenzhen Liu 2022	154.031	111.851	32	137.172	64.783	29	12.3%	16.86 [-28.50, 62.22]
**Total (95% CI)**			**399**			**546**	**100.0%**	**-6.43 [-22.32, 9.46]**

Heterogeneity: Chi² = 1.19, df = 2 (P = 0.55); I² = 0%
Test for overall effect: Z = 0.79 (P = 0.43)

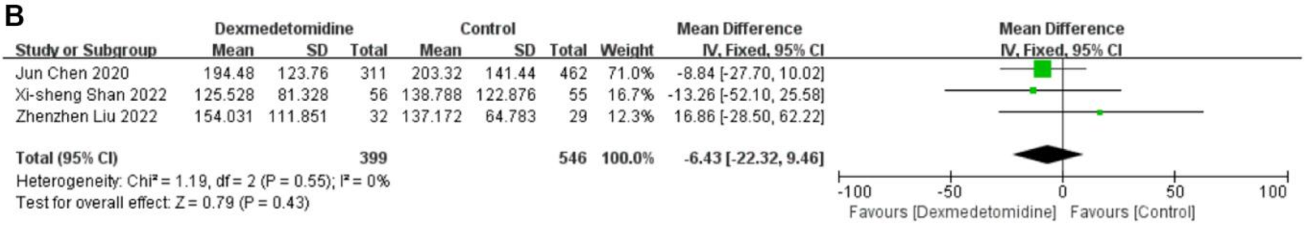

C

Study or Subgroup	Dexmedetomidine Mean	SD	Total	Control Mean	SD	Total	Weight	Mean Difference IV, Fixed, 95% CI
Jin Ha Park 2021	100.776	39.78	51	111.384	26.52	52	51.6%	-10.61 [-23.69, 2.47]
Jun Chen 2020	150.28	106.08	310	150.28	79.56	456	45.8%	0.00 [-13.88, 13.88]
Zhenzhen Liu 2022	153.875	158.994	32	130.862	49.771	29	2.6%	23.01 [-34.98, 81.00]
**Total (95% CI)**			**393**			**537**	**100.0%**	**-4.87 [-14.26, 4.53]**

Heterogeneity: Chi² = 2.10, df = 2 (P = 0.35); I² = 5%
Test for overall effect: Z = 1.02 (P = 0.31)

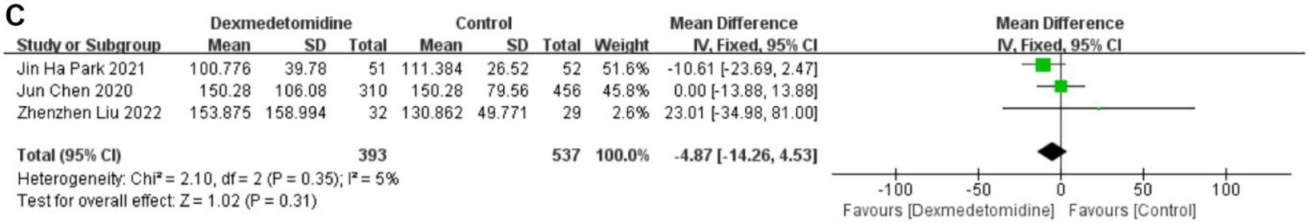

Supplementary Figure 2. Forest plots of the effect of DEX on creatinine level at 7 days (**A**), 1 month (**B**) and 3 months (**C**) after RT.

A

Study or Subgroup	Dexmedetomidine Mean	SD	Total	Control Mean	SD	Total	Weight	Mean Difference IV, Fixed, 95% CI
Jun Chen 2020	53.1	26.5	311	51.8	26.3	462	67.2%	1.30 [-2.50, 5.10]
Xi-sheng Shan 2022	65.1	24.1	56	63.3	27.4	55	10.5%	1.80 [-7.81, 11.41]
Zhenzhen Liu 2022	33.23	12.9	31	35.05	13.16	29	22.3%	-1.82 [-8.42, 4.78]
**Total (95% CI)**			**398**			**546**	**100.0%**	**0.66 [-2.46, 3.77]**

Heterogeneity: Chi² = 0.71, df = 2 (P = 0.70); I² = 0%
Test for overall effect: Z = 0.41 (P = 0.68)

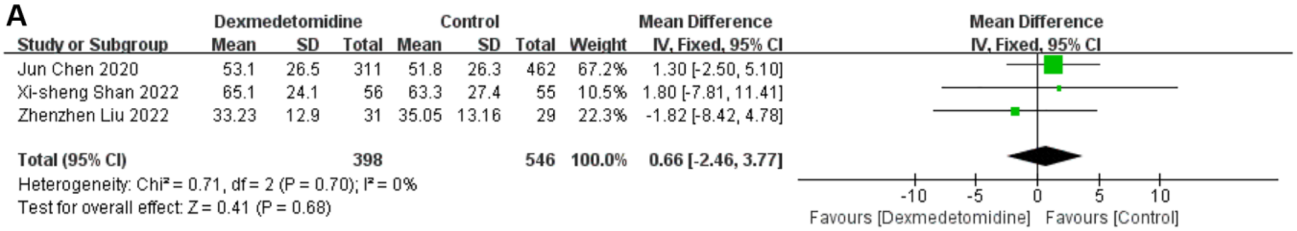

B

Study or Subgroup	Dexmedetomidine Mean	SD	Total	Control Mean	SD	Total	Weight	Mean Difference IV, Fixed, 95% CI
Jin Ha Park 2021	66.85	19.72	51	67.19	16.38	52	17.0%	-0.34 [-7.35, 6.67]
Jun Chen 2020	63.8	24.7	310	64.2	26.4	456	62.3%	-0.40 [-4.06, 3.26]
Zhenzhen Liu 2022	35.4	12.23	31	35.8	12.84	29	20.7%	-0.40 [-6.75, 5.95]
**Total (95% CI)**			**392**			**537**	**100.0%**	**-0.39 [-3.28, 2.50]**

Heterogeneity: Chi² = 0.00, df = 2 (P = 1.00); I² = 0%
Test for overall effect: Z = 0.26 (P = 0.79)

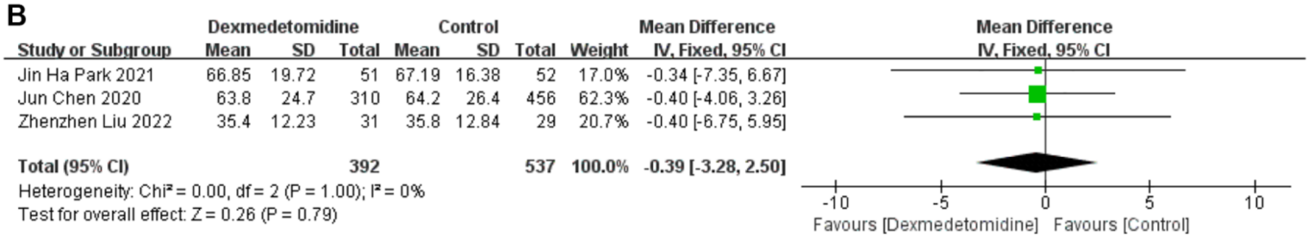

Supplementary Figure 3. Forest plots of the effect of DEX on eGFR at 1 month (**A**) and 3 months (**B**) after RT.
