# Supplementary material for: The positive efficacy of dexmedetomidine on the clinical outcomes of patients undergoing renal transplantation: evidence from meta-analysis
Source: Aging (Albany NY). 2023 Dec 11;15(23):14192–209. doi: 10.18632/aging.205296 (PMC10756127; doi:10.18632/aging.205296)
Supplement: Supplementary Table 1 [file aging-15-205296-s002.pdf]

SUPPLEMENTARY TABLE

Supplementary Table 1. The search strategy of this study.

Database	Search strategy
The Cochrane Library	#1 MeSH: [Kidney Transplantation]<br>#2 (renal or kidney) AND (transplant* or graft*)<br>#3 #1or#2<br>#4 MeSH descriptor: [Dexmedetomidine]<br>#5 MeSH descriptor: [Adrenergic alpha-Agonists]<br>#6 (Precedex or Dexmedetomidin*) or ((adren?ergic or alpha) near agonist*)<br>#7 #4or#5or#6<br>#8 #3and#7
MEDLINE (PubMed)	#1 Kidney Transplantation/<br>#2 (renal or kidney) AND (transplant* or graft*)<br>#3 #1or#2<br>#4 Dexmedetomidine/<br>#5 Adrenergic alpha-Agonists/<br>#6 Precedex or Dexmedetomidin*<br>#7 (adren?ergic or alpha) adj3 agonist*<br>#8 #4or#5or#6or#7<br>#9 #3and#8
EMBASE	#1 Kidney Transplantation/<br>#2 (renal or kidney) AND (transplant* or graft*)<br>#3 #1or#2<br>#4 Dexmedetomidine/<br>#5 Adrenergic alpha-Agonists/<br>#6 Precedex or Dexmedetomidin*<br>#7 (adren?ergic or alpha) adj3 agonist*<br>#8 #4or#5or#6or#7<br>#9 #3and#8
https://www.clinicaltrials.gov/	#1 kidney transplantation<br>#2 Dexmedetomidine
